# Development and Integration of a Solar Powered Unmanned Aerial Vehicle and a Wireless Sensor Network to Monitor Greenhouse Gases

**DOI:** 10.3390/s150204072

**Published:** 2015-02-11

**Authors:** Alexander Malaver, Nunzio Motta, Peter Corke, Felipe Gonzalez

**Affiliations:** 1 Institute for Future Environments, School of Chemistry, Physics and Mechanical Engineering, Queensland University of Technology, 3 George St, Brisbane 4000, Australia; E-Mails: alexander.malaverrojas@qut.edu.au (A.M.); n.motta@qut.edu.au (N.M.); 2 School of Electrical Engineering, Computer Science, Robotics and Autonomous Systems, Queensland University of Technology, Brisbane 4000, QLD, Australia; E-Mail: peter.corke@qut.edu.au; 3 Australian Research Centre for Aerospace Automation (ARCAA), Queensland University of Technology, Brisbane Airport, QLD 4007, Australia

**Keywords:** air pollution monitoring, environmental monitoring, gas sensors, greenhouse gases, nanostructured metal oxide sensors, UAV, UAV, WSN, solar energy

## Abstract

Measuring gases for environmental monitoring is a demanding task that requires long periods of observation and large numbers of sensors. Wireless Sensor Networks (WSNs) and Unmanned Aerial Vehicles (UAVs) currently represent the best alternative to monitor large, remote, and difficult access areas, as these technologies have the possibility of carrying specialized gas sensing systems. This paper presents the development and integration of a WSN and an UAV powered by solar energy in order to enhance their functionality and broader their applications. A gas sensing system implementing nanostructured metal oxide (MOX) and non-dispersive infrared sensors was developed to measure concentrations of CH_4_ and CO_2_. Laboratory, bench and field testing results demonstrate the capability of UAV to capture, analyze and geo-locate a gas sample during flight operations. The field testing integrated ground sensor nodes and the UAV to measure CO_2_ concentration at ground and low aerial altitudes, simultaneously. Data collected during the mission was transmitted in real time to a central node for analysis and 3D mapping of the target gas. The results highlights the accomplishment of the first flight mission of a solar powered UAV equipped with a CO_2_ sensing system integrated with a WSN. The system provides an effective 3D monitoring and can be used in a wide range of environmental applications such as agriculture, bushfires, mining studies, zoology and botanical studies using a ubiquitous low cost technology.

## 1. Introduction

Large scale monitoring of gases produced by the environment, industry and agriculture is a demanding task that requires long periods of observation, large numbers of sensors, data management, high temporal and spatial resolution, long term stability, computational resources, and energy availability. WSNs and UAVs are a good alternative for such demanding tasks, although their development and availability is limited by factors such as sensor stability over long periods, energy availability when deployed in remote areas, payload weight for small Unmanned Aerial Vehicles (UAVs), management of data produced by sensor nodes, and cost.

Recent technological improvements in gas sensors, electronics, telecommunication, solar cells, and avionics have made possible the development of WSNs and UAVs equipped with gas sensing systems for high spatial and temporal resolution. Such systems have broad scientific and industrial applications including monitoring anthropogenic emissions of greenhouse gases (GHG) such as CO_2_ [1,2], as well as local pollutants from bushfires, cities, factories, and agricultural fields such as NO_2_ [3,4] and CH_4_ [5–7]. Practical application of WSNs has been proposed for monitoring fugitive CH_4_ emissions [6], coal fields or biomass degradation (landfills) [7], and NH_3_ and N_2_O gas releases from fertilizer use [8,9]. Some of these systems are already commercially available, but the cost/benefit ratio is still too high to be widely used. WSNs are essential to monitor large areas such as cities, roads, and forests due to their ability to communicate via nodes and multi hop data. These functionalities for example may allow the tracking and mapping of gas plumes to identify the plume origin at ground level.

UAVs can play an important role in environmental gas sensing in remote areas due to their capability to carry instruments, sensors and collect data with high spatial and temporal resolution [10,11]. UAVs have already been used to this purpose; for instance, Watai *et al.* [12] reported on the development of a non-dispersive infrared (NDIR) sensing system on a small UAV to monitor atmospheric CO_2_ concentrations. The authors designed and built an economic and accurate gas sensor system (±0.26 ppm precision) and performed several flight tests with a one hour flight autonomy and 3.5 kg payload. McGonigle, *et al.* [13] reported the measurements of volcanic gases with a helicopter UAV at La Fossa crater, Volcano (Italy), using an ultraviolet and infrared spectrometer to measure SO_2_ and CO_2_ gas concentrations. This UAV had a 3 kg payload weight and 12 min flight autonomy. Astuti, *et al.* [14,15] developed a fixed wing UAV for volcanic monitoring at Mt Etna. The UAV carried a CO_2_ infrared spectrometer and an SO_2_ electrochemical sensor. Khan, *et al.* [16] developed a greenhouse gas analyser using a vertical cavity surface emitting laser (VCSELs) embedded in a helicopter UAV. CO_2_, CH_4_ and water vapour were targeted by developing a sensing module for each targeted gas, with a vertical and horizontal resolution of less than 1 m. Each module weighed 2 kg and required 2 W of power to operate.

The most popular gas sensing technology used in WSNs for environmental monitoring is based on Metal Oxide (MOX) resistive sensors, while optical gas sensing devices are more popular among UAV users [6,7,9,12,13,17]. This research aims at using the same sensing technology to integrate WSNs and UAVs in order to reduce complexity and cost. Both technologies were evaluated according to their advantages and disadvantages for ground and aerial mission; monitoring of continuous and instant release of pollutants; computational resources required; maximum achievable resolution; and financial cost. Table 1 presents a comparison of these two technologies.

Recent advances in nanotechnology have benefited the development of MOX sensors facilitating the synthesis of novel classes of materials with enhanced gas sensing performance [18]. Within this nano-range, the physical, chemical, optical, mechanical, electronic and biological properties of these materials can be substantially different from those observed for the bulk sensing materials [18,19]. Such unique properties are attributed to the small size, as the quantum regime becomes predominant over the classical limit. The performance of nano-structured sensors depends also on the type of morphology. Research on 1-D nanostructures for gas sensing applications has intensified due to their high surface-to-volume ratio, quantum confinement and improved crystallinity [20]. The most common 1-D metal-oxides nanostructures used in the fabrication of resistive based gas sensors are in the form of nanorods, nanowires, nanofibers, nanotubes, nanobelts, nanoribbons, nanowhiskers, nanoneedles, nanopushpins, fibre-mats, urchin, lamellar and hierarchical dendrites [21]. The MOX nanowires demonstrated improved sensitivity to a wide range of gas species and stability due to their high degree of crystallinity [22]. The increasing number of scientific publications focused on nanowires and nanowire based sensors during the last ten years reached its highest peak in 2011 [23]. The addition of a small amount of noble metals like Ag, Au, Pd, and Pt over the MOX surface; tuning of the working temperature toward a given compound with respect to another, coating the surface by specific functional groups can increase up to five times their sensitivity [24]; and multi-component sensing elements (sensor array) coupled with signal processing functions can be applied to differentiate the response of nanowires toward the target gas [22,25]. MOX sensors have found wide spread commercial applications [26], and most WSN users employed commercial MOX sensors. For the above mentioned reasons, this research selected MOX nanowires to measure CH_4_ concentrations. Since optical sensing technologies have been widely tested for gas sensing applications and have produced high quality and reliable results [27] a NDIR sensor device was also selected to measure CO_2_ concentrations.

Power is major issue for portable applications such as WSN and UAV because its availability limits their service life, reduces data collection and limits its applications. WSNs powered by solar energy have been developed [28,29], but their use for environmental gas sensing is limited. The concept of harvesting solar energy to power aircrafts in the field of UAVs has a long history and many solar powered aircraft have been successfully created [30,31]. The UAV developed in this work pursuit flight endurance and the ability to power a gas sensing system simultaneously. The following section describes the development of the gas sensing technology developed for the WSN and UAV.

## 2. Sensing System Design

### 2.1. Solar Powered WSN

**Figure 1.**
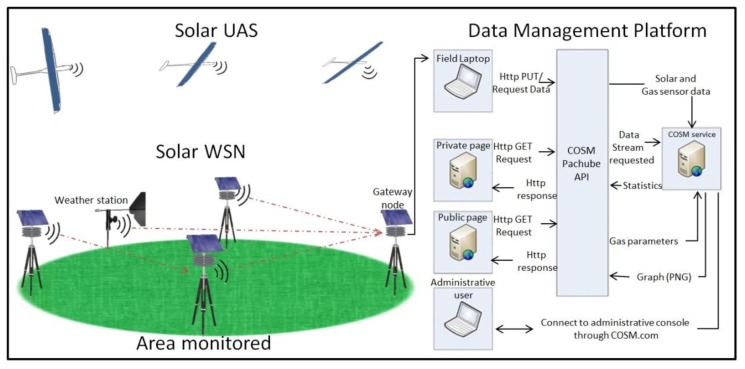
Illustrates the design of a solar powered WSN and a UAV integrated to a data management platform for continuous monitoring of pollutant gases.

**Figure 2.**
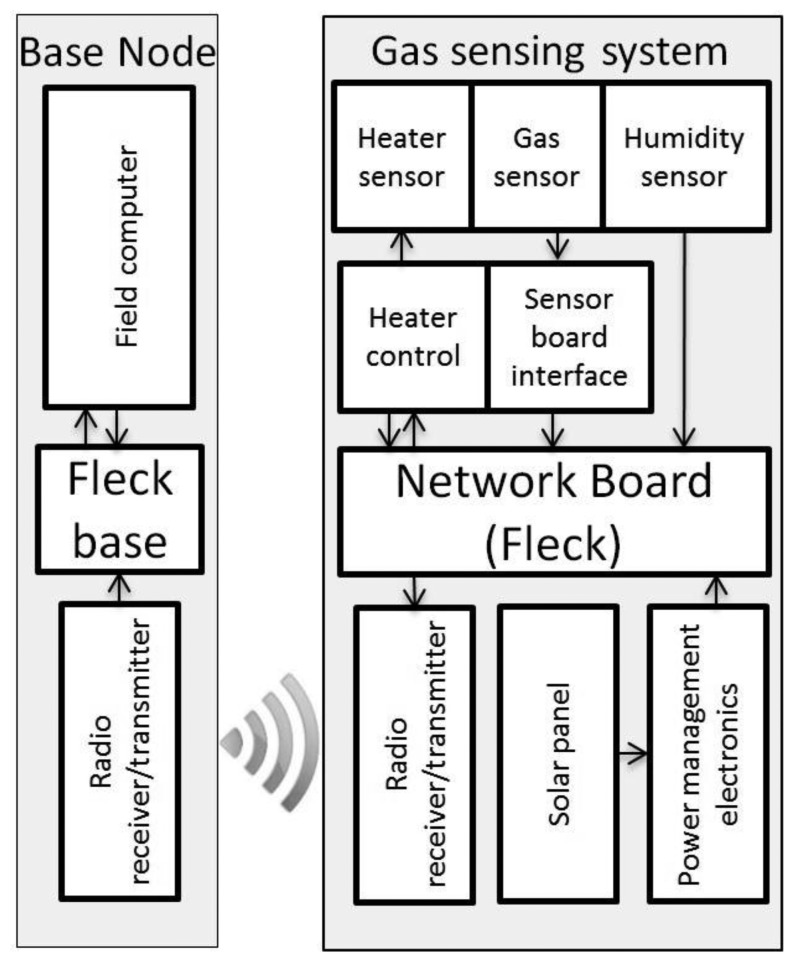
Wireless sensor node and base node configuration.

The four principal components of our wireless sensor node are the Fleck [32,33], which is a microprocessor with networking capabilities, the humidity sensor, the solar panel with its power management electronics and the gas sensing system. The WSN was created by using the Fleck network cards developed by CSIRO [32,33]. The sensor board interface, microprocessor and communication capabilities of the sensor node were tested and reported in previous research papers [34–36]. Data collected from sensor nodes were stored and displayed on live webpages using the data management platform illustrated in Figure 1. The base node (Figure 2) was equipped with a Fleck and connected to the field computer by USB.

*Humidity Sensor*: humidity has an important influence on the performance of gas sensors, especially MOX sensors. Water absorbed on the MOX surface will not donate electrons to the sensing layers, lowering the MOX sensitivity [20]. Prolonged exposure of sensors to humid environments leads to the gradual formation of stable chemisorbed OH^−^ on the surface causing a progressive deterioration of the performance of gas sensors [20]. Humidity interference is not expected in the SnO_2_ sensor used for CH_4_ samples, as the sensor temperature was higher than 200 °C, where molecular water is no longer present at the surface [37]. For this reason, the sensor node was equipped with a humidity sensor for studies where the sensor is kept at temperatures below 200 °C. A HIH-4010 humidity sensor from Honeywell Inc. (Minneapolis, MN USA) was selected as this sensor produces an output voltage (∼0.8–3.8 V) proportional and linear to the humidity percentage. The sensor board reads this signal using one of the ADC ports, and the data acquired is used to compensate any drift in the sensor baseline or sensor response, when the sensor works below 200 °C.

*Solar Panel and Power Management*: the power electronics manage the energy provided by the solar panel to supply regulated power to the sensor node (3.5−6 V, 500 mA), recharge a standard lithium battery (3.7 V, 1200 mAh) with the surplus energy, and keep the solar panel working at the maximum power point (MPPT). The BQ 24030 electronic chip from Texas Instruments Inc. (Dallas, TX, USA) [38] was selected and configured to develop this task, which bench testing and results were reported in previous published papers [34–36].

### 2.2. Gas Sensing System for the Wireless Sensor Network

*MOX sensor*: several MOX sensors developed by Brescia University and QUT (Queensland University of Technology) were tested at different CH_4_ concentrations. Laboratory results indicated that a tin oxide (SnO_2_) nanowire was the best candidate to be implemented in the WSN due to its appealing characteristic among the developed MOX sensors [25,39]. MOX sensors usually require working temperatures between 150 °C and 400 °C to activate the chemical reactions leading to the resistivity change when interacting with gases. The fabricated sensor has an embedded platinum heater at the back of the sensor plate. After several outdoor experiments, it was found that the sensor baseline drifted due to environmental conditions such as humidity and correlation to other gases [40]. A drifty baseline affects the reliability of the sensor measurements and requires re-calibration procedures. This undesirable effects increase substantially in aerial applications due to higher wind speed and variable atmospheric conditions.

In response to this challenge, a heating cycle protocol was developed to stabilize the sensor baseline for outdoor performance. The sensor heater was connected in series to a high frequency switching transistor and a shunt resistor to control and measure the delivered power. The transistor collector was connected to the positive power, the gate was connected to the Fleck and the Source was connected to the sensor. The power was provided by a fixed DC voltage (3.3 V), which was applied to the transistor. The opening and closing time of the transistor gate was controlled by the Fleck with a Pulse Width Modulation (PWM) signal, which regulates the heater current. The duty cycle of the PWM signal was adjusted automatically from 50% to 100% to reach the selected temperature based on the current measured on the shunt resistor, which is in series with the heater sensor. The heating cycle of the sensor was set to 300 °C for 2.5 min, when exposed to CH_4_ concentrations; and 400 °C for 2 min in air, after each gas measurement to evaporate any water or gas molecule attached to the sensor surface, which produced a stable baseline.

**Figure 3.**
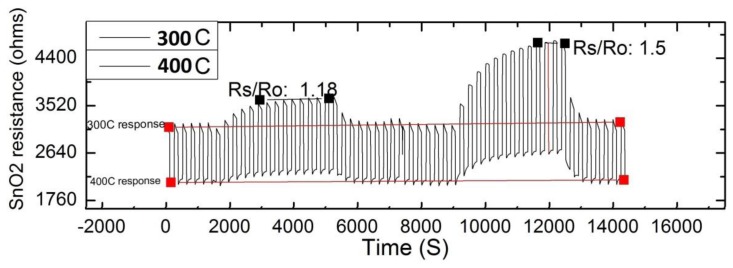
Response of the SnO_2_ sensor for different concentrations of CH_4_. The cycling temperature of the heater was 300 °C for 2.5 min and 400 °C for 2 min.

*MOX-CH_4_ gas testing*: the sensor response to CH_4_ concentrations was evaluated at QUT laboratories by using a high precision multi-channel gas testing system. The testing system includes a 1100 cc test chamber capable of testing four sensors in parallel, eight high precision mass flow controllers (MKS 1479A, Andover, MA, USA) to regulate the gas mixture, 8-channel MFC processing unit (MKS 647C), and a picoammeter (Keithley 6487, Cleveland, OH, USA). The measurements were performed with a mixture of synthetic air and CH_4_ gas at different concentrations (up to a maximum of 10.6 ppm of CH_4_ balanced in synthetic air), 25 °C, and 0 humidity. The right concentrations of CH_4_ gas in air were obtained by adjusting the respective flow rates via the MFCs, while maintaining a total constant flow rate of 200 SCCM (mL/min). The sensor heater was connected to an electronic board that executed the heating protocol described previously, and the picoammeter applied 1 bias volt to the sensor upon gas exposure in order to read the sensor resistance. The sensor was left in the test chamber overnight under dynamic flow of synthetic air, which helps to stabilize the sensing layer before the test. Once the sensor was stable, it was tested towards 5 and 10.5 ppm of CH_4_ for 5 times in order to characterize the sensor response. The average time response (t_r_) of the sensor was 15.7 min and 24.3 min, respectively when exposed to 5 ppm and 10.5 ppm of CH_4_; and the average recovery time was 8.7 min at 5 ppm, and 8.86 min at 10.5 ppm. This sampling frequency will suit most of the studies required for ground pollutants, however this response time will hinder aerial applications that require faster responses. The sensor response can be expressed as the ratio of R_s_/R_o_, where R_s_ is resistivity in gas and R_o_ is the resistivity in air. R_s_ varied from 0 to 1.5, when CH_4_ concentrations varied from 0 to 10.5 ppm, respectively (Figure 3). These values show that the sensor has high sensitivity to the gas for a short concentration span.

**Figure 4.**
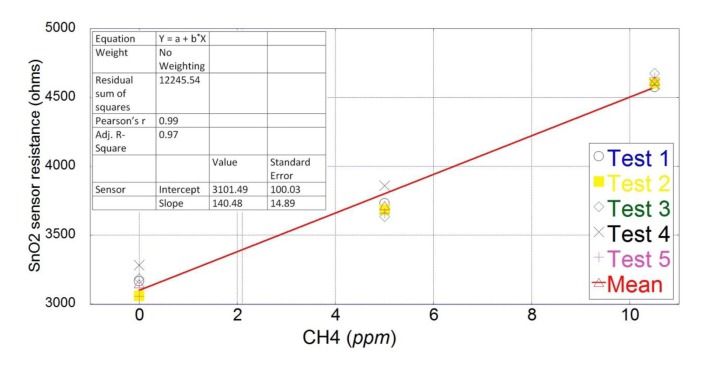
Linearization of the sensor response in resistance towards CH_4_ concentrations from 0 to 10.5 ppm.

The sensor response exhibits a linear behavior for this short span concentration according to Figure 4. Therefore, the sensor response as a change in resistance was linearized to estimate concentrations from 0 to 10.5 ppm. The independent variable (gas concentration) was plotted on the *X* axis, while the dependent variable (estimated concentration) was plotted on the *Y* axis. A simple linear regression or least mean square (LMS) model was applied to the data in order to calibrate the system.

The intercept term (*q*) and slope parameter (*m*) were calculated using the following equations [41]:

(1) Y=mX+q+ε(1)

where *m* was calculated by using Equation (2):

(2) $$\text{m}=\frac{(\text{N}\sum_{\text{i}}\left({\text{X}}_{\text{i}}\times {\text{Y}}_{\text{i}}\right)-\left(\sum_{\text{i}}\left({\text{X}}_{\text{i}}\right)\times \left(\sum_{\text{i}}{\text{Y}}_{\text{i}}\right)\right)}{\text{N}\sum_{\text{i}}{\left({\text{X}}_{\text{i}}\right)}^{2}-{\left(\sum_{\text{i}}{\text{X}}_{\text{i}}\right)}^{2}}$$

and *q* was calculated by using Equation (3):

(3) $$\text{q}=\frac{\left(\sum_{\text{i}}{\text{Y}}_{\text{i}}\right)\times \sum_{\text{i}}{\left({\text{X}}_{\text{i}}\right)}^{2}-\left(\sum_{\text{i}}{\text{X}}_{\text{i}}\right)\times \left(\sum_{\text{i}}\left({\text{X}}_{\text{i}}{\text{Y}}_{\text{i}}\right)\right)}{\text{N}\sum_{\text{i}}{\left(\text{X}\right)}^{2}-{\left(\sum_{\text{i}}{\text{X}}_{\text{i}}\right)}^{2}}$$

The variables are defined as:

*X*: known gas concentration
*Y*: sensor response in resistance (ohms)
*N*: total experimental points
*i*: sequence of each experimental point

By replacing the values on equation 1, the new estimated gas concentration (*Y*) values were defined as:

(4) Y=3101.4+140.48X

**Figure 5.**
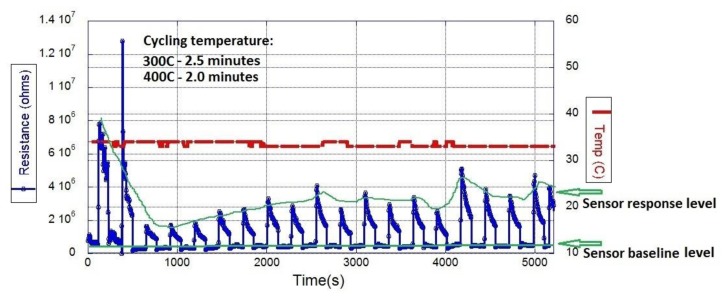
SnO_2_ sensor response towards environmental gases in SERF, QUT, Brisbane, Australia.

*Field testing of the sensing systems*: the baseline stability of the SnO_2_ sensor was tested outdoors on the roof of S block at QUT, Gardens Point and at SERF (Samford Ecological Research Facility), QUT. The sensor was placed in a special sensor shelter powered by a solar panel. The temperature of the sensor was controlled by the heating protocol described in previous section, which results are plotted in Figure 5. Two sensor response levels are clearly identified from the graph in Figure 5. The bottom level was produced by the sensor response when heated for 2 min at 400 °C*.* This sensor response level was stable, with almost no drift and was used as sensor baseline. The top level response, produced when the sensor was heated for 2.5 min at 300 °C, is the sensor response towards environmental gases.

*CO_2_module*: CO_2_ concentrations were measured by an off-the-shelf NDIR sensor (CDM30K, Figaro Inc., Osaka, Japan), which is pre-calibrated from factory at 0 and 400 ppm*.* The accuracy of the reading were cross checked with a LI-840A CO_2_ analyzer for one operational day showing an overall error in the measurements of 5%. The signal output of the module is a DC voltage between 0 and 4 V, which represents 0–2000 ppm, respectively [42].

**Figure 6.**
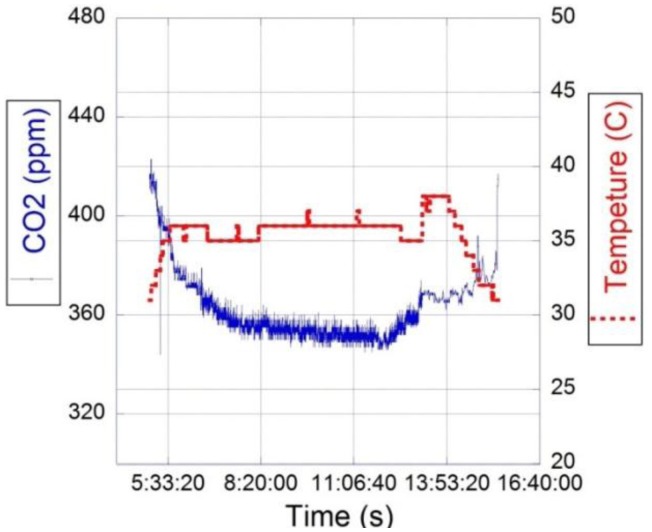
Response of one of the CO_2_ nodes installed at SERF (QUT, Brisbane, Australia) during one day of operation.

The sensing system was tested under different environmental conditions in a farm field for 93 days. Figure 6 shows the CO_2_ concentration and the temperature registered by one of the sensor modules during one day of operation. The CO_2_ concentrations were mostly influenced by vegetation activity of the surrounded rural area, which increased the CO_2_ levels during night periods and decreased it during sun-light hours. Conversely, the environmental temperature presented the opposite behavior. The carbon dioxide levels registered are similar to the values recorded by George *et al.* [43] in rural areas with extensive vegetation. Additionally, the output data was cross checked periodically with a CO_2_ analyzer (Li-840A) to verify the reliability of the readings. Significant loss in performance was not detected during the time span of the experiment.

### 2.3. Gas Sensing System for the Solar Powered UAV

**Figure 7.**
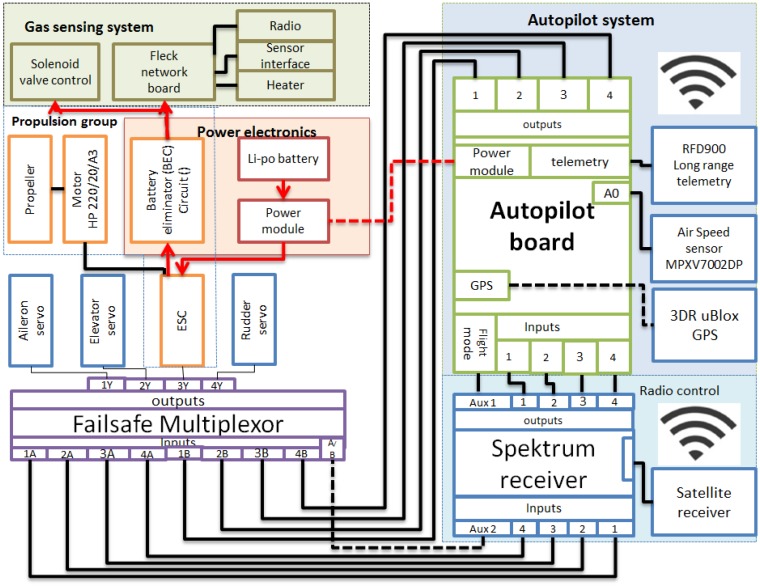
Configuration of the four main sub-systems integrated in the UAV.

The main sub-systems of the UAV are the gas sensing, navigation, communication, propulsion and power system, which are highly integrated into the aircraft frame. The block diagram of each sub-system is depicted in Figure 7. The Gas Sensing System described in Section 2.2 was installed in the UAV, and a sampling system was adapted to the sensor due to higher wind speed conditions. The main components of the system are the sensor, sensor heater, sensor board interface, a network board (Fleck) with radio transmitter/receiver capability and a solenoid valve control.

**Figure 8.**
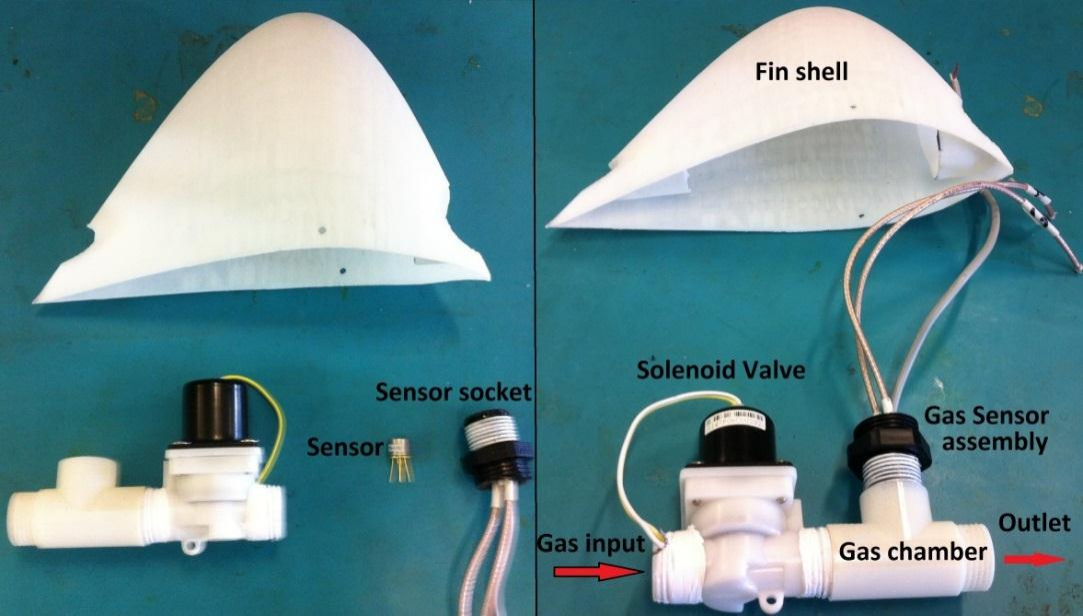
Gas sensing system for airborne applications: aerodynamic fin shell, gas sensor, sensor socket, gas chamber and solenoid valve.

The adaptations performed on the gas sensing system to make it functional for aerial missions are illustrated in Figure 8. The sample intake was adapted to capture samples for gas analysis during flight maneuvers. A fin shell was designed and 3D printed to house the gas sensing system on top of the central wing. The fin shell was made of lightweight materials (<50 g) to avoid significant drag and weight to the aircraft. A small gas chamber (63 cm^3^) was designed and installed inside the fin shell to retain the sample volume during analysis. The gas chamber has a T shape to let the sample flows across the horizontal trajectory and to insert the sensor in the vertical cavity, which ensures proper contact with the gas volume (Figure 8). A solenoid valve was installed at the inlet of the chamber to control the time and flow of the sample intake (Figure 8). The closing time of the valve depends on the sensor response time to the expected gas concentration, for instance 5 s close was enough time to analyze CO_2_ concentrations from 0 to 400 ppm; and 2 s was the opening time of the valve to completely flush the chamber after each analysis.

The valve was closed or opened by changing the polarity of the solenoid inductor, which requires a power pulse of 6 V/580 mA for at least 30 m. The cycling time of the sampling system could be controlled by the Fleck microprocessor which activated the solenoid depending on the gas concentration detected. A second control option was an electronic timer circuit (LM 555), which time was fixed before the mission started. The electronic battery eliminator circuit (BEC) of the Electronic Speed Controller (ESC) of the aircraft provided up to 5 VDC, 2 A of power for the gas sensing system, and a step-up converter circuit attached to the BEC provided the 6 V required to activate the solenoid valve. Once the Fleck acquired the sensor data, the information was transmitted to the base node by using the radio module of the Fleck, antenna of which was installed on the top of the airframe.

**Figure 9.**
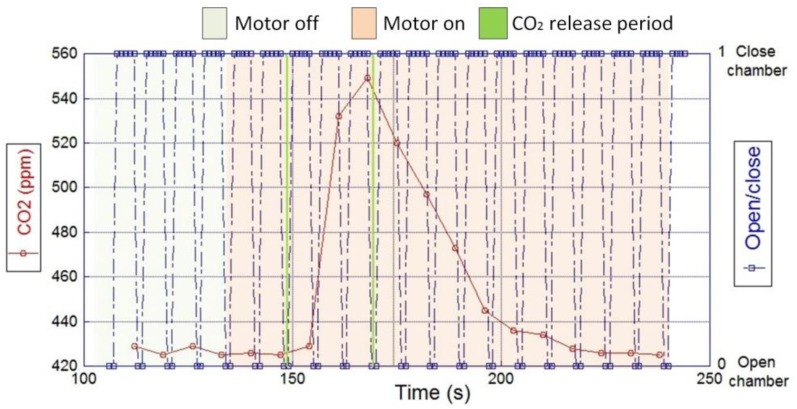
Bench testing of the CO_2_ gas sensing system integrated in the aircraft fuselage.

*Bench and field testing of the Gas Sensing System for the UAV*: the CO_2_ sensing system was mounted inside the fin shell, which was installed on top of the middle wing. A bench test was conducted with the engine, propeller and avionics of the aircraft running during the emission of a pollutant source. Figure 9 graphs the response of the CO_2_ module during the experiment, which shows that the sensing system successfully detected a CO_2_ peak within 60 s after the pollutant emission started; it shows that the sensor baseline was not altered by the downstream wind produced by the propeller, and the sensor baseline returned to its original level of about 425 ppm after the emissions stopped. The background level registered by the sensor corresponds to the CO_2_ concentration of the surrounded volume.

Another bench testing was conducted to evaluate the performance of the CH_4_ system integrated to the UAV. The development of the experiment was similar to the previously described CO_2_ test; except for the valve control that was manually activated to determine the response time of the SnO_2_ sensor. The testing procedure was developed as follows (Figure 9):

- First, the stability of the sensor baseline was verified with the avionics and motor of the aircraft switched off.
- Then, CH_4_ emissions were released from a pollutant source in front of the UAV for 12 min, until the sensing system started to register changes in the sensor resistance.
- Next, the motor was switched on, clearing any remainder of emissions inside the chamber. It was observed that the sensor baseline dropped back to the resistance level registered at the beginning of the test.
- Emissions from a contaminant source were continuously released for 34 min at a rate of 1 L/min, while the motor was kept at 50% power.
- Once the emissions reached the gas sensing system, the solenoid valve was closed to fill the chamber with the gas volume, and let the sensor response to the gas concentration.
- After the sensor response was stable, the solenoid valve was re-opened for 2 s to flush the chamber, producing a sudden decrease in the sensor resistance.
- The previous procedure was repeated twice to verify the functionality of the solenoid valve and the sensor response to the contaminant.

**Figure 10.**
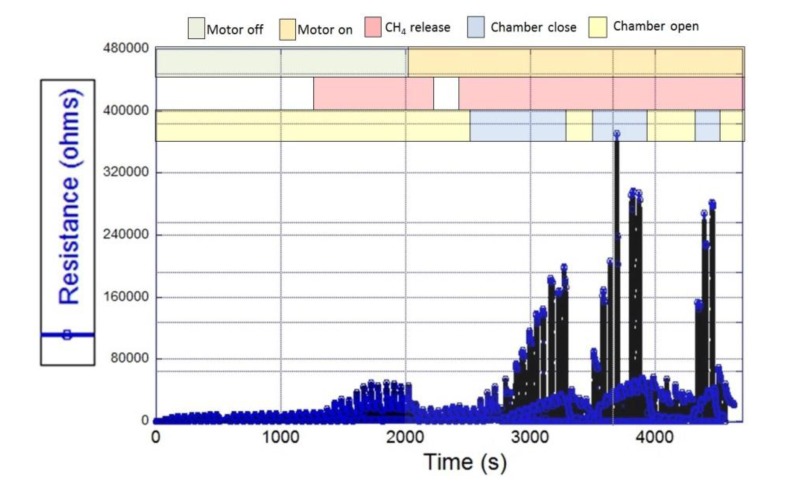
Bench testing of the CH_4_ sensing system with a pollutant emission source.

The experiment successfully tested the performance of the gas sensing system during an emulated airborne operation, which results are plotted in Figure 10. The sensor baseline was stable under the regular wind flow produced by the natural atmospheric dynamics and when the propeller was activated during the experiment, indicating a noise free background of the system. After the chamber was closed the sensing system detected variations in the sensor resistance, which indicates a successful capture of the sample and a stable environment inside the chamber. The closing time of the valve allowed the sensor to reach its chemisorption and physisorption stability on each measurement. When the solenoid opened the chamber, the sample was washed away producing a sudden decrease in the sensor resistance, until it reached its baseline level again. It was observed that the probability of detecting contaminants in front of the aircraft was increased due to the vortex effect of the propeller. This fact confirmed that the location of the gas sample intake was not negatively affected by the propeller, which on the contrary could have a beneficial effect.

## 3. Solar UAV Design and Flight Test

The UAV developed in this work was based on the Green Falcon UAV [44,45] developed at QUT and the Australian Research Centre for Aerospace and Automation (ARCAA). The principal sub-systems are: (*i*) Navigation system, which main components are the autopilot, air speed sensor, gyro sensor, accelerometer, magnetometer, barometric pressure, GPS, and fail safe system. The autopilot used in the UAV was the ArduPilot Mega 2.5, which is a complete open source autopilot system with a high benefit/cost ratio [46] and low weight (23 g). The autopilot system works mainly in three modes: autonomous mode, to fully perform unmanned mission by pre-programing waypoints from the ground control station (GCS). Stabilized mode, to assist a ground pilot in controlling and stabilizing the flight of the aircraft; in this mode the pilot has partial control of the aircraft and when there is no pilot input the autopilot will maintain a level flight of the aircraft. Manual mode, which is useful to perform the pre-flight check as the autopilot acts as a pass-through for all the RC commands; this mode allows the pilot to freely preform manual take-offs, maneuvers and landings, when the autopilot is not pre-programed to perform these tasks. In all modes, the autopilot is capable of transmitting important flight information such as roll, pitch, yaw, airspeed, GPS position and battery status to the GCS by using the telemetry module.

The telemetry module used was the RFD900, which works at 900 MHz, is lightweight (50 g), small size, has large transmission range (>40 km), and requires about 1 W (+30 dBm) transmit power. The Mission Planner GCS was selected to create the waypoint mission based on Google maps, send commands to the autopilot, receive and graph in real time autopilot's data outputs, download mission log files, and data analysis.

**Figure 11.**
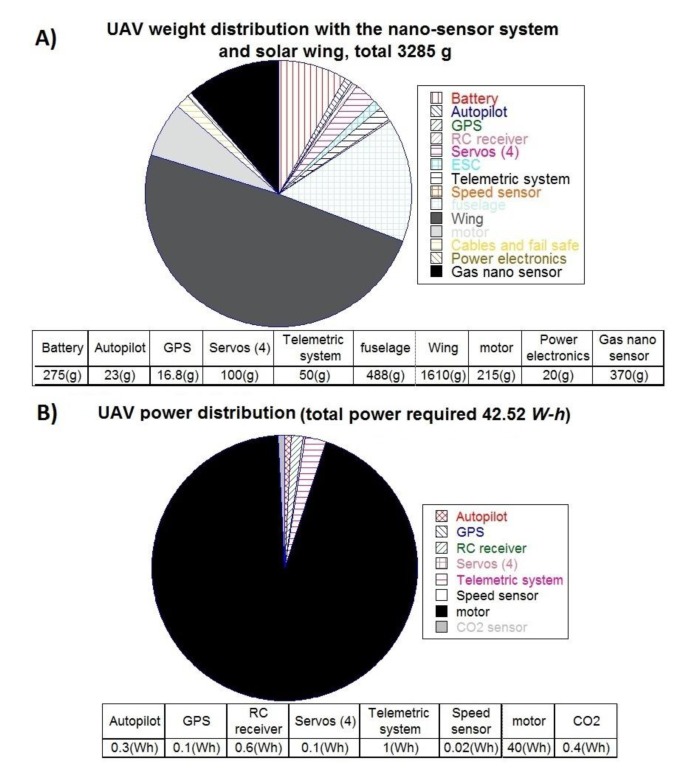
(**A**) Weight distribution of the UAV with the nano-sensor system; (**B**) power consumption breakdown of the UAV assembled with the CO_2_ sensing system.

The airframe is easy to transport for fast deployment and hand launched take off; it has a wingspan of 2.52 m, wing aspect ratio (AR) of 13, and length of 960 mm. The original weight of the wing was 960 g, and after the addition of the SSC panels it increased to 1610 g; therefore, the final weight of the UAV was 3285 g. (Figure 11A). The net power consumption of the UAV was 42.52 Wh, when equipped with the CO_2_ sensing system (Figure 11B), and 42.92 Wh with the nano-sensor system. The pie chart evidenced that the power consumption of the gas sensing system was only a small proportion of the total energy demand; and the energy consumption does not vary significantly between the CH_4_ and CO_2_ sensing systems.

The total energy demand of the UAV is expected to be higher due to electronics inefficiencies that are calculated using Equation (5):

(5) $${\text{E}}_{\text{demand}\_\text{total}}=\frac{\left({\text{E}}_{\text{avionics}}+{\text{E}}_{\text{gas}\_\text{s}}\right)}{\eta_{\text{power electronics}}\times \eta_{\text{avionics}}}$$

where the efficiency of the power electronics (*η_pe_*) is 0.86, and the avionics (*η_av_*) is 0.90.

Replacing values in Equation (6):

$$\begin{array}{c} {\text{E}}_{\text{demand}\_\text{total}}=\frac{\left(42.12Wh+0.8Wh\right)}{0.86\times 0.9}\\ {\text{E}}_{\text{demand}\_\text{total}}=55.4Wh \end{array}$$

The total energy demand (55.4 Wh) needs to be supplied by the solar wing and the battery as follows.

**Figure 12.**
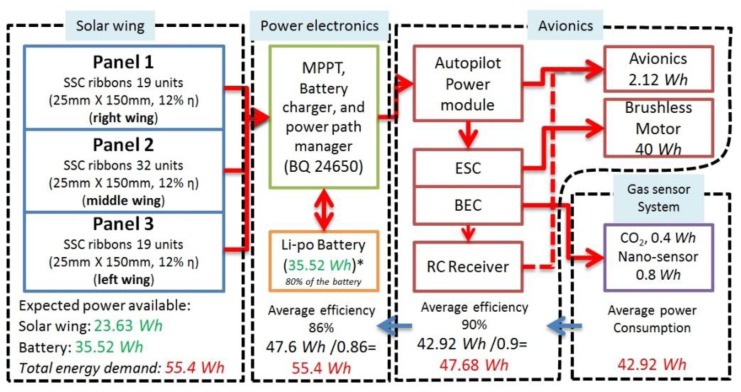
Energy demand and the energy available in the UAV.

The solar panels for the wing were constructed using small silicon solar cells (SSC) ribbons connected in serial and parallel configuration to achieve the voltage and current required. Each SSC ribbon has an area of 0.00375 m^2^ and 12% efficiency. The solar panel area was limited by the wing area (490 cm^2^), ailerons, narrow ends, and the area allocated for the gas sensing system (53 cm^2^). Finally, 70 SSC ribbons were distributed along the available wing area (Figure 12), which output power produced was calculated as follows:

(6) $${\text{Area}}_{\text{SSC}\_\text{panel}}={\text{Area}}_{\text{SSC}\_\text{ribbon}}\times 70\text{units}=0.2625m^{2}$$

The average output energy of the panels was calculated based on the mean sunshine hours of Brisbane (QLD, Australia), which are 7.4 h with a mean irradiance of 750 Wh/m^2^, according to the Australian Bureau of Meteorology. Therefore, the expected average energy produced by the solar wing is:

(7) $${\text{Energy}}_{\text{SSC panel}}=\frac{0.2625m^{2}\times 750Wh\times 0.12}{1m^{2}}=23.625Wh$$

A commercial lithium polymer 44.4 Wh, 3.0 mAh 4 cells battery was used in combination with the solar panel to meet the energy demand of the aircraft. Only 80% of the battery capacity (35.52 Wh) was taken into account for safety reasons. The total energy expected (Energy_SSC panel_ + Energy_battey_) was therefore 59.14 Wh, enough to satisfy the total energy demand of the UAV. Figure 12 summarizes of the energy demand of the UAV and the energy available.

**Figure 13.**
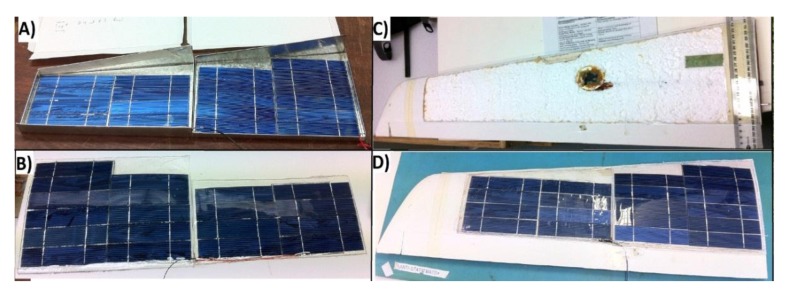
Solar powered wing construction: (**A**) left wing SSC panel in the mold; (**B**) right wing SSC panel encapsulated with clear and flexible resin; (**C**) left wing peeled on the skin to accommodate the solar panel; (**D**) final installation of the SSC panel on the surface of the wing.

The SSC ribbons were distributed along the three parts of the wing by placing 19 units on each side wing (total 38), and 32 units in the middle wing for a total of 70 SSC units (0.2625 m^2^). The weight density of a single SSC ribbon with the tabbing wire installed was 0.53 kg/m^2^, and the internal connections of panels were in serial configuration. The SSC panels were encapsulated in a flexible structure that takes the shape of the wing to avoid losses in aerodynamic performance and to withstand mechanical stress produced by the aircraft in operation. The encapsulation was developed by using a clear resin, which is flexible and totally transparent (Figure 13).

**Figure 14.**
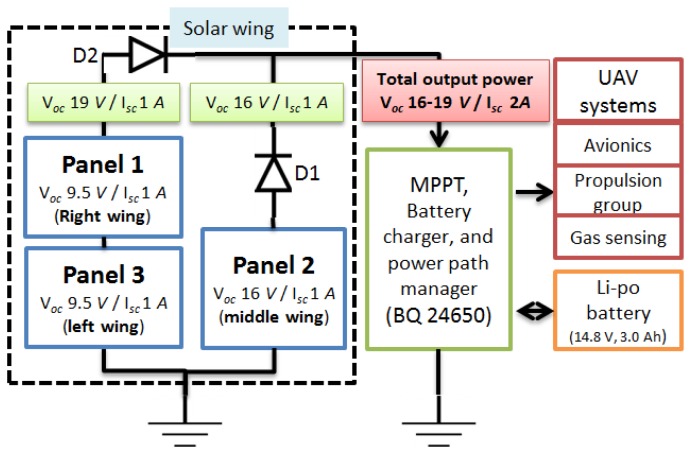
Panel configuration for the solar wing.

The open circuit voltage (V_oc_) of the side wing panels was 19 V_oc_, with an expected short circuit current (I_sc_) of 1.16 A. The middle wing panel was constituted of 32 SSC ribbons in serial configuration, which produced 16 V_oc_, and short circuit current (*I_sc_*) of 1.16 A (Figure 14). The right and the left wing panels were connected in series, and the output of these were connected in parallel to the middle wing panel in order to produce a final output of about 16–19 (V_oc_), 2 A (I_sc_) (Figure 14). The panels have slightly different output voltage was due to space limitation in the wings; this fact is not desirable because it produces two different maximum power points and the panel with lower voltage becomes a load for the other. The problem can be solved by using two MPPT at the outputs, at expenses of increasing the power consumption and weight of the aircraft. For this reason, the solution implemented was to set the maximum power point of the MPPT in the middle of both output voltages (17 V) to mitigate this effect; this solution is viable due to the proximity of both output voltages. During flight operations is likely that part of the solar panel area is shaded due to flight manoeuvres; if this is the case, the shaded panel becomes a load for the others panels connected in parallel, or an obstruction for panels connected in series. A diode was installed at the output of each panel to create a bridge or a bypass to avoid this negative effect (Figure 14).

## 4. Field Testing of the Gas Sensing Technology and UAV

**Figure 15.**
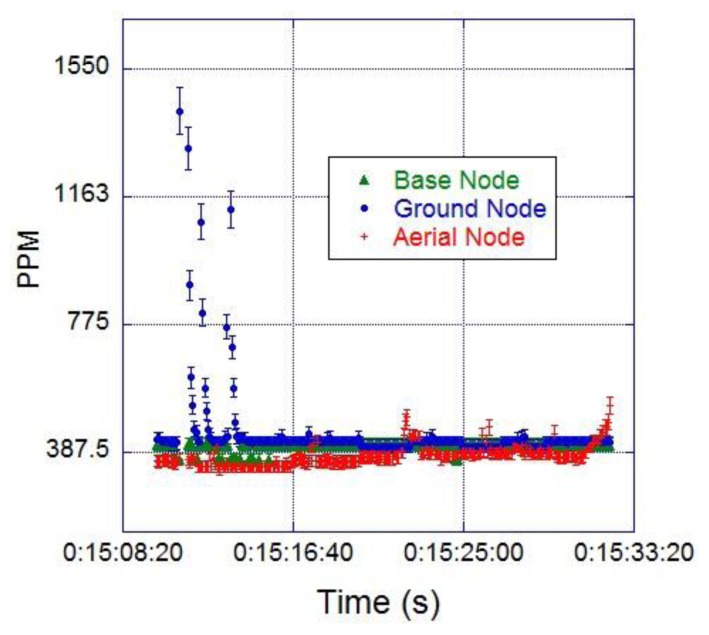
Monitoring of atmospheric CO_2_ integrating two ground nodes and one aerial node.

The first field test involved two nodes to monitor CO_2_ at ground level, and the UAV equipped with the original wing (non-solar) to monitor CO_2_ at about 100 m ASL. One ground node was connected to the base computer and the other solar powered node was located 30 m away, south of the ground station. The mission consisted of a continuous circular flight above the ground nodes for 20 min. The CO_2_ readings recorded from the three sensors are shown in Figure 15. The geo-location of each sample was not reported in this experiment as the autopilot or a separate on-board GPS was not involved in the experiment. The graph shows that the ground node and the base node registered similar CO_2_ concentrations of about 399 ppm throughout the test; the node located 30 m away showed some CO_2_ spikes at the beginning of the test, corresponding to the emulation of a contaminant source. The aircraft readings are represented by the red line and their average value was 379.7 ppm. The readings from the UAV were slightly lower than the readings registered by the ground nodes, probably due to higher wind speed and slightly lower atmospheric pressure experienced during the flight mission. The values obtained are reasonable in comparison to the 392.722 ppm of CO_2_ recorded by the Cape Grim Baseline Pollution Station (Tasmania, Australia), at the atmospheric baseline in June 2013.

**Figure 16.**
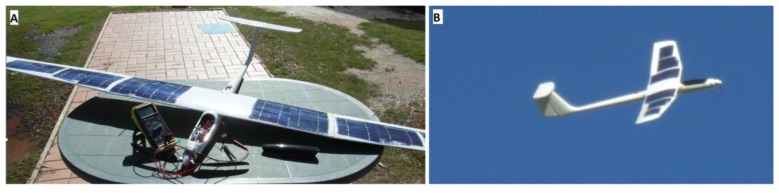
(**A**) Final configuration of the solar UAV; (**B**) aerial photography of the aircraft flying.

The second test evaluated the solar powered wing and power electronics. The solar wing was installed on the aircraft and produced an open circuit voltage (V_oc_) of 20.7 V, given the sun irradiance conditions of that day before the flight. The short circuit current (I_sc_) was 2 A at full sun irradiance. The UAV was hand launched with the solar wing and the flight lasted for 20 min, showing a stable performance of both the aircraft and wing structure. Post-landing inspections did not show any significant deformation or structural failure of the solar panel or wing shape, indicating a successful construction technique for the solar wing. Figure 16 shows the final configuration of the solar UAV before the flight and an aerial photography of the aircraft flying.

The final test integrated a GCS, a base node, one CO_2_ ground node, a weather station, and the solar UAV equipped with a CO_2_ node. The field test was developed in Christmas Creek, QLD on the 23 July 2013 and included the controlled release of a contaminant source. The CO_2_ ground node and the weather station were deployed 20 m away, and the pollutant source 30 m away, south of the CGS. The mission of the UAV was to fly in a circular trajectory up to 50 m ASL over the area monitored, especially above the sensor node and pollutant source. The CO_2_ contaminant was release for 6 min, at a rate of 0.0027 kg/s, for a total mass of 1 kg with an average wind speed of 1.09 m/s (Figure 18).

**Figure 17.**
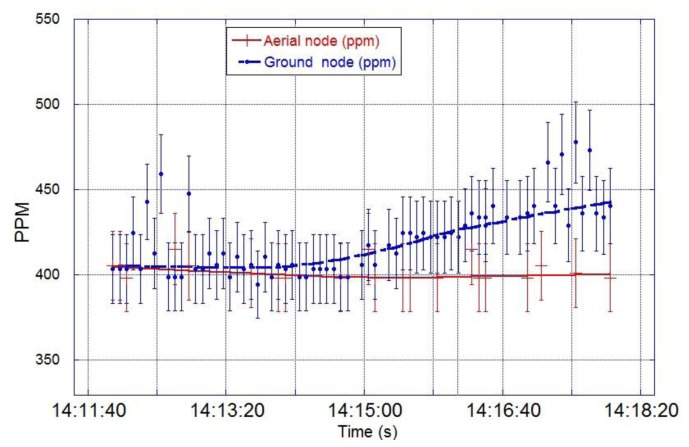
CO_2_ readings from the ground node and aerial node during the field testing at Christmas Creek, QLD the 23 July 2013.

The flight operation lasted for 20 min, and the base node successfully collected data from the ground and aerial nodes simultaneously. Figure 17 shows that the average CO_2_ concentration registered by the ground node was 404 ppm during the first 164 s; then, the concentration rose to an average value of 442 ppm when the contaminant was released. The average CO_2_ concentration registered by the aerial node was 400 ppm during the whole test, with few CO_2_ peaks above the average. The field experiment was designed based on Papanikolaou *et al.* [47] studies on short term release of CO_2_ from the Kit Fox gas field experiments. Their simulations showed that release of CO_2_ clouds from natural sources or Carbon Capture and Storage (CCS) places can reach concentrations higher than 100.000 ppm with volumes of more than 100 m^3^ in few seconds. In the UK, the limits of CO_2_ work place exposure are 0.5% (5000 ppm) for long term exposure (8 h); 1.5% (15,000 ppm) for short term exposure (15 min); and 70,000–100,000 ppm for instant exposure (>1 s), which represents immediate danger to human life or health. The proposed WSN and UAV are suitable to perform monitoring on previous scenarios were ground sensors can raise an early alarm that launches a UAV to monitor the evolution of the CO_2_ cloud. The data collected will be invaluable for emergency service and control systems in order to evacuate affected areas and predict the evolution of the pollutant cloud.

**Figure 18.**
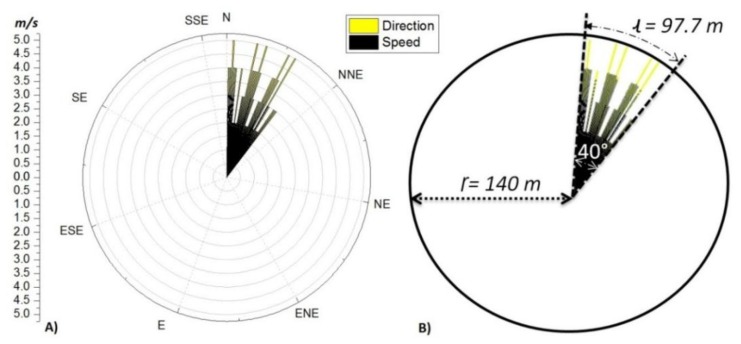
(**A**) Illustrates the direction and speed of the wind during the field testing; (**B**) illustrate the monitored area affected by the pollutant emissions due to the wind.

Post-flight analysis on the data collected from the GCS logs indicated that the horizontal sampling resolution of the UAV was 88.2 m, based on the average cruise speed of 12.6 m/s and the sampling frequency of 7 s. The total volume monitored was 3 × 10^6^ m^3^ based on the circular area travelled ((Γ) = 140 m) by the UAV and the flight altitude of about 50 m ASL. Figure 18 illustrates the area monitored by the UAV, where the origin of the circle represents the contaminant source and the area affected by the emissions is delimited by *l*, due to the wind effect. The wind was blowing constantly in the North-East direction during the test, creating a narrow corridor of about 40° (ɵ) for the contaminant emissions.

Therefore, the maximum radio (Γ) of the monitored area was 140 m, with a maximum arc length of 97.7 m. This indicated that just one measurement was possible per circular flight on the affected zone due to the horizontal resolution of the UAV (88.2 m).

**Figure 19.**
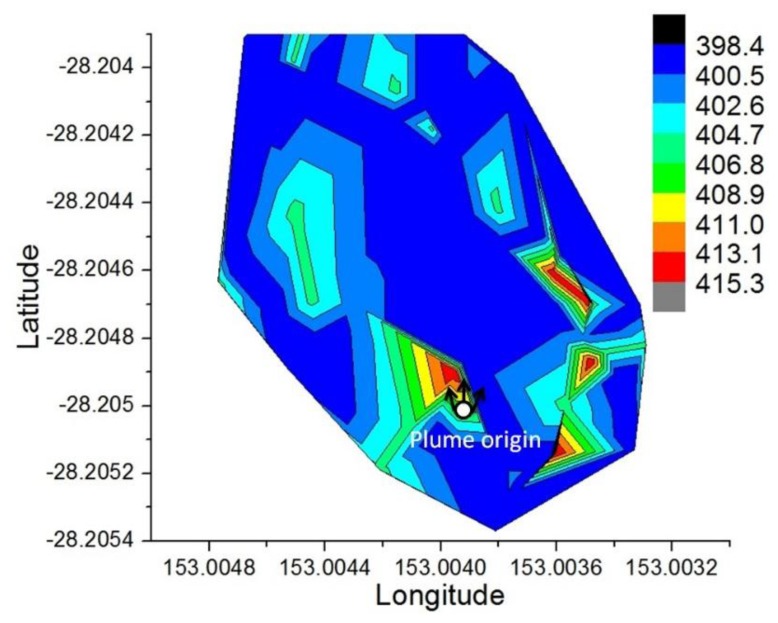
Contour map of the CO_2_ concentration estimated by the UAV within the 3 × 10^6^ km^3^ monitored.

Geo-location of the sample was possible by synchronizing the log's time of the gas sensing board and the autopilot GPS before the mission started. The ability to geo-locate the sample and register the time allowed reconstruction of taken samples in three dimensions, which facilitates visualization of local concentrations and analysis. The data collected allowed the creation of contour maps that help to identify gradients of concentrations within the volume monitored. The CO_2_ data values were interpolated based on Renka [48] and Yuan [49] algorithms used in OriginPro Graph processor program. The methodology creates contour maps in four steps: Triangulation, Linear Interpolation, drawing of contour lines and smoothing of the curves, based on the average flight altitude (50 m ASL), gas concentration, longitude and latitude coordinates of taken sample (Figure 19).

**Figure 20.**
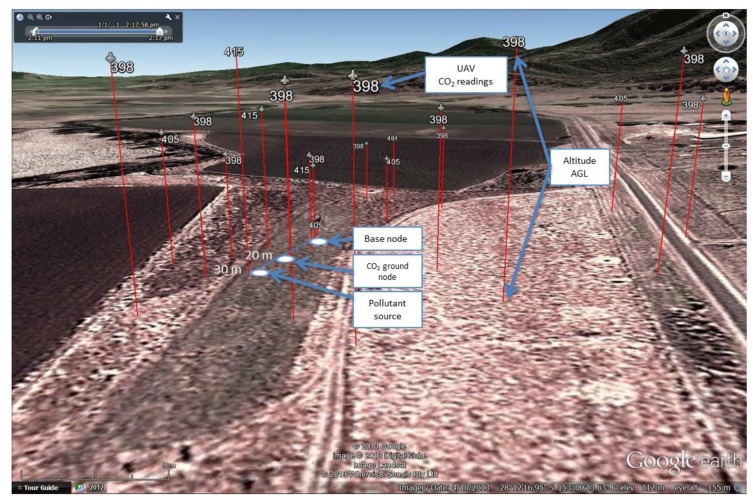
Geo-location and values of the CO_2_ samples taken by the UAV during the field testing in Christmas Creek, 23 July 2013.

A 3D map in Google Earth was created based on the geo-location of the taken samples. Figure 20 indicates the real position of the base node, ground node, pollutant source and the taken samples during the experiment. A video of the project development is online at the Green Falcon project channel [50].

## 5. Conclusions

The WSN, UAV and gas sensing systems developed in this research are a response to challenges and limitations of WSNs and UAVs in the field of gas sensing and energy availability. The successful integration of a small solar powered aircraft equipped with a gas sensing system and networked with solar powered ground nodes proves the possibility of 3D monitoring of pollutant gases.

Our electric powered aircraft allowed the use of sensitive instruments and the execution of circular trajectories without self-contamination. A sensing system based on resistive MOX sensors was evaluated both for the WSN and the UAV. We addressed the problem of the drifty baseline caused by environmental humidity and correlation to other gases by implementing a heating cycling protocol of the sensor. However, MOXs sensors were not used in flight operations due to their long response time that hinder aerial applications. Further research and development of MOXs nano-sensors is required to achieve the detection of single molecules of gas instantaneously [18]. Both our resistive gas sensing system and a commercial NDIR module were successfully adapted and tested for aerial missions, showing reliable performance and meeting the payload constraints of a small aircraft. A method to design, create and evaluate small solar powered UAVs equipped with a gas sensing system was successfully developed and validated with experimental testing.

The results are significant, as we believe that this prototype system is an important step for the future of environmental monitoring; and the advances in solar cells, batteries, and sensing technologies will open a wide market of intensive and capillary environmental data acquisition, not limited only to gas concentrations, but also to temperature, humidity, aerosols, pollens, *etc.*

## Figures and Tables

**Table 1.** Advantages and disadvantages of MOX and optical gas sensing technologies when used in WSNs and UAVs.

**Category**	**MOX Sensors**		**Optical Sensing Techniques**	
	**Advantages**	**Disadvantages**	**Advantages**	**Disadvantages**
Aerial missions	Low energy consumption and light weight	Slow sensor response hinder aerial applications	Tested and proved	Energy consumption and weight may limit flight endurance
Ground missions	Tested and proven	Cross reference to different gases and sensitive to humidity	High sampling frequency, high specificity to target gas	No literature found, sensor are too expensive to be left unattended
Continuous release mission	Low energy and light weight, covers wide range of gases	No literature found	High sampling frequency, high specificity to target gas	Energy consumption and weight may limit flight endurance
Instantaneous release	Low energy and light weight, cover wide range of gases	Low sensor response time	High sampling frequency, high specificity to target gas	Energy consumption and weight may limit flight endurance
Computational resources	Few output variables, and same variables remain over large range of gases	No literature found -	No literature found	The number of output variables to measure depends on the optical technique used and target gas
Resolution	Regular resistive sensors achieve *ppm* resolution	Few sensors achieve *ppb* resolution	Several techniques achieve *ppm* and *ppb* resolution	No literature found
Cost position in Market cost	Low	None	NDIR modules have Low	Complex systems Medium to High
